# Regenerative Effects of Hypoxia Primed Flowable Placental Formulation in Muscle and Dermal Injury

**DOI:** 10.3390/ijms22137151

**Published:** 2021-07-01

**Authors:** Sandeep Dhall, Min Sung Park, Chaoyang Li, Malathi Sathyamoorthy

**Affiliations:** Smith & Nephew Plc., Columbia, MD 21046, USA; zhaoyli@gmail.com (C.L.); msathyam39@hotmail.com (M.S.)

**Keywords:** amnion, chorion, umbilical cord, ischemia, chronic wounds

## Abstract

The placental tissue, due to its angiogenic, anti-inflammatory, antioxidative, antimicrobial, and anti-fibrotic properties, has become a compelling source towards a solution for several indications in regenerative medicine. However, methods to enhance and capture the therapeutic properties with formulations that can further the applications of viable placental tissue have not been explored. In this study, we investigated the regenerative effects of a hypoxia primed flowable placental formulation (FPF), composed of amnion/chorion and umbilical tissue, in two in vivo injury models. Laser Doppler data from rodent ischemia hindlimbs treated with FPF revealed significant tissue perfusion improvements compared to control ischemic hindlimbs. To further corroborate FPF’s effects, we used a rodent ischemic bipedicle skin flap wound model. FPF treatment significantly increased the rate of wound closure and the quality of wound healing. FPF-treated wounds displayed reduced inflammation and an increase in angiogenesis. Furthermore, quantitative PCR and next-generation sequencing analysis confirmed these changes in the FPF-treated group at both the gene and transcriptional level. The observed modulation in miRNAs was associated with angiogenesis, regulation of inflammatory microenvironment, cell migration and apoptosis, reactive oxygen species generation, and restoring epithelial barrier function, all processes involved in impaired tissue healing. Taken together, these data validate the tissue regenerative properties of the flowable placental formulation configuration tested.

## 1. Introduction

Tissue regeneration generally involves the process of regenerating, replacing, or repairing damaged or defective cells, tissues, and/or organs in the body to restore their normal function. Examples of regenerative medicine include cell-based therapies, biologically active molecule-based therapies, and tissue engineering. Mesenchymal stromal cells (MSCs) derived from either the placental membranes comprising the amnion and chorion or the umbilical cord have exhibited therapeutic regenerative functions, including angiogenic, anti-inflammatory, antioxidative, antimicrobial, and antifibrotic properties, when used in cell-based therapies [1,2]. Methods to enhance the regenerative functions of the MSCs prior to use have been studied using in vitro models of cell priming [3]. These methods involve isolating the MSCs from tissue either by enzymatic digestion or other methods and can further involve the passaging of the isolated cells to expand the cell number. The isolated/expanded cells are then exposed to hypoxic conditions, UV light, culture conditions, incubation, or other cell priming conditions [4]. However, the process of isolating and expanding the MSCs can reduce their stemness and other therapeutically beneficial properties. Moreover, other therapeutically beneficial native cells, such as fibroblasts and epithelial cells, that may be present within the tissue are lost during the isolation of the MSCs. Thus, cell-based therapies containing primed MSCs may have limited therapeutic effects.

Amnion, chorion, and the umbilical cord contain extracellular matrix (ECM) that house viable native cells (including MSCs, fibroblasts, and epithelial cells), cytokines, growth factors, and other nutrients, making them a potent solution for a variety of indications in regenerative medicine. Placental tissues are currently used in the management of indications in wound treatment, orthopedics, sports medicine, ear/nose/throat (ENT), trauma, dental, and other fields where regenerative properties such as the angiogenic, anti-inflammatory, antioxidative, antimicrobial, and antifibrotic properties of placental tissues are extremely beneficial. However, methods to enhance or boost the reservoir of therapeutic regenerative properties of native placental tissue before further downstream processing have yet to be explored. 

An initial priming of placental membranes and the umbilical cord by subjecting it to environmental stresses could result in enhancing in situ cellular response, including increased production of growth factors, antimicrobial peptides, cytokines, extracellular vesicles, exosomes, extracellular matrix (ECM), and other bioactives, all of which are indicated for successful tissue repair and regeneration. Given the difficulties of tissue repair and regeneration in various disease states and chronic injury indications, a multifactorial approach is warranted. 

We hypothesized that subjecting the placental membrane and umbilical cord to hypoxia preconditioning would enhance the production of responsive bioactive factors by the viable cells in the tissue. Using the hypoxia primed placental membranes and umbilical tissue, we generated a microparticulate flowable formulation. We determined the regenerative effects of this flowable placental formulation (FPF), which consisted of the primed placental membranes (amnion and chorion) and primed umbilical cord, in both in vitro bioassays and in vivo models of chronic injury conditions. To test the hypothesis, we used the well-established rodent model for hindlimb ischemia and the rodent model for bipedicle flap ischemic wounds. In the hindlimb ischemia model, we observed increased hindlimb perfusion upon treatment with FPF. Similarly, we determined increased rate and quality of wound healing in FPF-treated animals. Furthermore, FPF-modulated gene and microRNA (miR) expression suggested transcriptional level changes that correlated with reduction in the inflammatory milieu. Taken together, these data validate the potential of FPF as a therapeutic modality that could be used in various clinical settings.

## 2. Results

### 2.1. Regenerative Properties of Hypoxia Primed FPF

**Figure 1:**
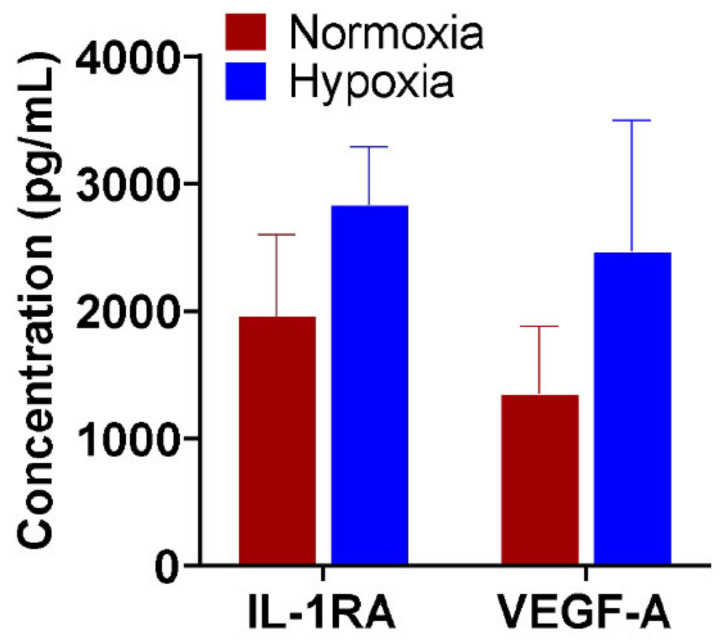
Hypoxia preconditioning enhances bioactive factors. Levels of IL1RA and VEGF-A were measured in placental membranes, amnion and chorion, and umbilical tissue in normoxia and after hypoxia conditioning. *N* = 3 donors.

**Figure 2:**
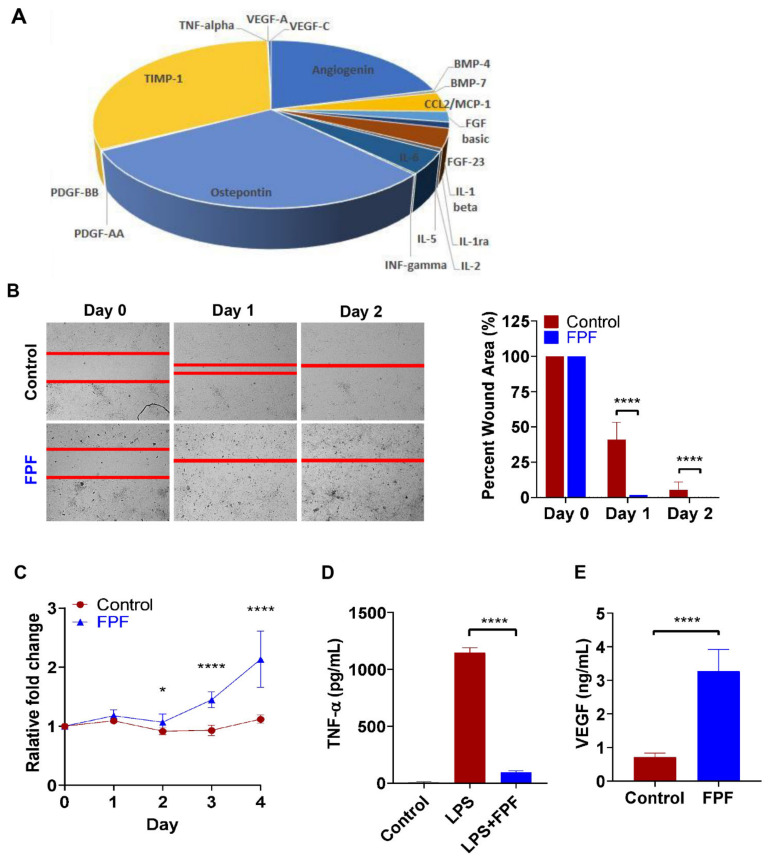
Tissue injury associated beneficial factors and their regenerative effects. (**A**) Representative presence of growth factors in FPF as determined by Luminex. *N* = 3 donors. (**B**) Representative photograph of HDF wound closure after incubation with FPF conditioned media. Graph shows significant reduction in wound area upon treatment with FPF conditioned media. Red line shows scratch gap closure (**C**) The viability of HDF cells was measured using MTT assay, and data are expressed as relative fold change compared to day 0. (**D**) Anti-inflammatory activity of FPF was evaluated by inhibition of TNFα release from stimulated human PBMCs. Unstimulated and stimulated PBMCs alone served as positive and negative controls, respectively. (**E**) Angiogenic activity of FPF was determined by a cell-based reporter assay that measured VEGF stimulation and inhibition of KDR (VEGFR-2) using luciferase as a readout. Results are shown as mean ± SD (*N* = 3). * *p* < 0.05, **** *p* < 0.0001.

The placental tissue in its native form has been shown to contain endogenous tissue regenerative properties [5,6]. To confirm whether hypoxia tissue priming resulted in enhanced tissue regenerative properties, we first evaluated the regenerative factors derived from the placental membranes (amnion and chorion) and umbilical cord after 48 h in a hypoxic incubator. We found that as a result of hypoxic priming, there was an overall increase in the secretions of IL-1RA, an anti-inflammatory factor, and VEGF, an angiogenic factor, as compared to tissues treated in normoxic condition (Figure 1). Since both the anti-inflammatory and angiogenic growth factor levels of the placental membranes were enhanced after hypoxia conditioning, we performed hypoxia conditioning of the amnion, chorion, and umbilical cord and prepared flowable placental formulation (FPF) using the primed tissue as starting material, via a proprietary process. We then assessed the growth factor and cytokine profile for FPF and confirmed the abundant presence of bioactive factors using multiplex analysis (Figure 2A). This plethora of factors present play a combinatorial role in tissue repair and regeneration.

As FPF is a microparticulate injectable formulation of placental tissues, using FPF conditioned media, we then assessed FPF for its effect on wound closure by performing an in vitro scratch wound healing assay (Figure 2B). HDF cells were cultured as a monolayer, and a wound field was created upon removal of the slide insert (Figure 2B, left panel). Cells were then incubated in FPF conditioned media and evaluated over a period of 2 days (Figure 2B, middle and right panel). We observed significant and complete wound closure by day 1 postincubation in FPF conditioned media as compared to control wounds (Figure 2B, middle panel). A significant difference in closure distance was also observed on day 2 after wound creation (Figure 2B, right panel).

The proliferation rate of HDFs was assessed by MTT assay (Figure 2C). HDFs treated with FPF exhibited significantly increased proliferation compared to the control by day 2. Although HDFs in the control group attached very well on the tissue culture plates at day 0, no significant increase in cell proliferation was observed during the 4-day incubation period in comparison to day 0 (Figure 2C). However, cell proliferation in the FPF-treated group significantly increased at both day 3 and day 4 compared to the control group.

To further corroborate the functionality of the bioactive factors, we carried out in vitro cell-based anti-inflammatory and angiogenic functional bioassays (Figure 2D,E). An overproduction in TNF-α, an inflammatory cytokine, in the injured tissue microenvironment prevents healing and tissue regeneration. Therefore, the anti-inflammatory capability of FPF was characterized by assessing the inhibition of TNF-α secretion in a bioassay with LPS-activated PBMCs. We show that FPF treatment significantly inhibited the production of TNF-α in LPS-activated PBMCs (Figure 2D). VEGF, another factor that plays a major role in tissue repair and angiogenesis, was assayed with both normoxic- and hypoxic-treated fractions of FPF using a VEGF-based reporter bioactivity system that comprised cells expressing VEGF-responsive luciferase. A significant increase in VEGF activity was observed in FPF prepared from primed tissue versus cells in normal growth medium (Figure 2E). Overall, these results suggest that FPF processed from hypoxia priming of human viable placental tissue was effective in enhancing the endogenous factors associated with regenerative functions.

### 2.2. FPF Increases Hindlimb Tissue Perfusion Upon Ischemic Tissue Injury

**Figure 3:**
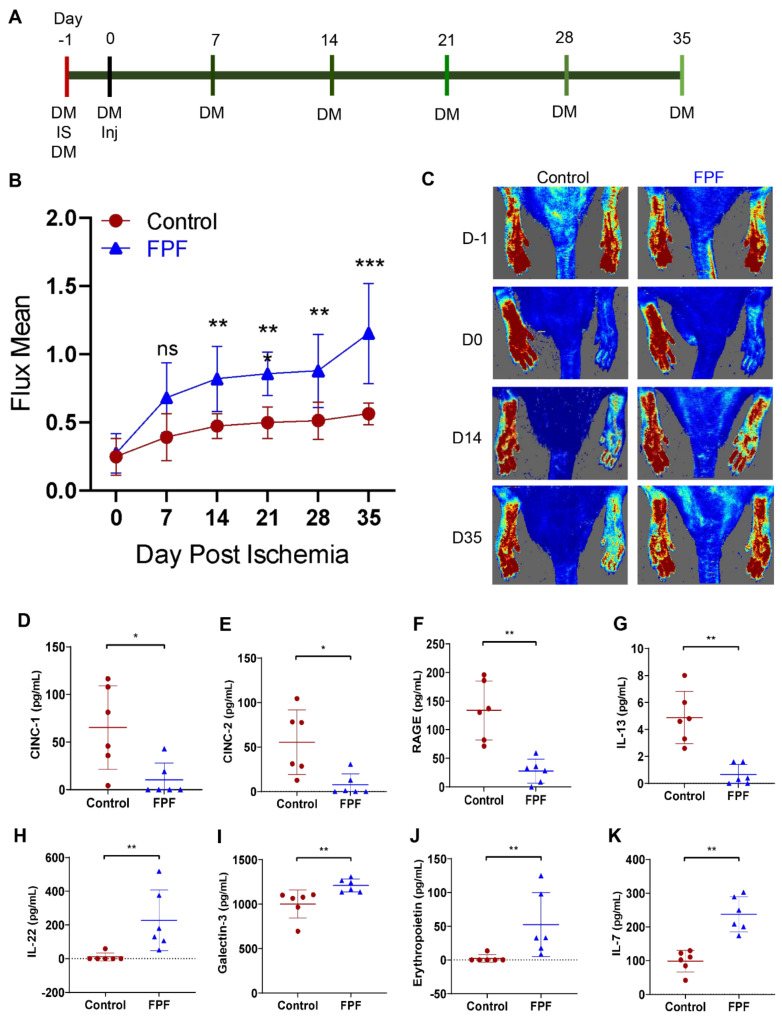
FPF restores tissue perfusion in a rat hindlimb ischemia model. (**A**) In vivo study timeline. DM = Doppler measurement, IS = ischemia surgery, Inj = FPF or saline injection. (**B**) Hindlimb perfusion measurements for animals treated with control or FPF. *N* = 10. Readings shown were taken after animals had been under anesthesia under imager for 10 min. ns: not significant. (**C**) Representative perfusion images for control (left panel) and FPF-treated (right panel) group after the animal was under anesthesia for 10 min at presurgery, the day of injection (1 day after ischemia induction), and day 35 post-treatment. Healthy limbs are on the left and ischemic/treated limbs are on the right. Levels of inflammatory and anti-inflammatory cytokines and chemokines in the Gracilis muscle at day 35 post-treatment for (**D**) CINC-1, (**E**) CINC2, (**F**) RAGE, (**G**) IL-13, (**H**) IL-22, (**I**) galectin-3, (**J**) erythropoietin, and (**K**) IL-7. N = 6 rats/group. * *p* < 0.05, ** *p* < 0.01, *** *p* < 0.005.

Peripheral arterial disease (PAD) is caused by the chronic reduction in perfusion and was mimicked using a rat hindlimb ischemia model (Figure 3). Vehicle control or FPF was injected a day after ischemia was induced, and tissue perfusion was measured using a High Resolution Laser Doppler Imager as shown in the study timeline (Figure 3A). This methodology resulted in a longitudinal assessment of full-field analysis of every rat. Percent flux of the ischemic limb to the healthy limb was calculated after the rats were stabilized under anesthesia for 10 min. The treatment with FPF resulted in significant increases in percent perfusion compared to the saline group as early as 14 days, and the significant improvement was maintained through the course of the study (Figure 3B,C). This shows that angiogenic and prohealing factors present in FPF stimulated an increase in hindlimb tissue perfusion that helped restore perfusion to normal levels.

To evaluate the effects of FPF in the hindlimb, we next sought to analyze the key cytokine biomarkers using a multiplex protein array in the Gracilis muscle after study completion at day 35. The analysis included cytokine-induced neutrophil chemoattractant-1 (CINC-1), cytokine-induced neutrophil chemoattractant-2 (CINC-2), receptor for advanced glycation end products (RAGE), interleukin-13 (IL-13), galectin-3, interleukin-22 (IL-22), and erythropoietin (Figure 3D–K). FPF-treated animals showed a significant decrease in proinflammatory factors (Figure 3D–G) and an increase in anti-inflammatory, proangiogenic, and ischemia protective factors (Figure 3H–K).

### 2.3. FPF Accelerates Ischemic Wound Healing and Closure with Improved Tissue Architecture

**Figure 4:**
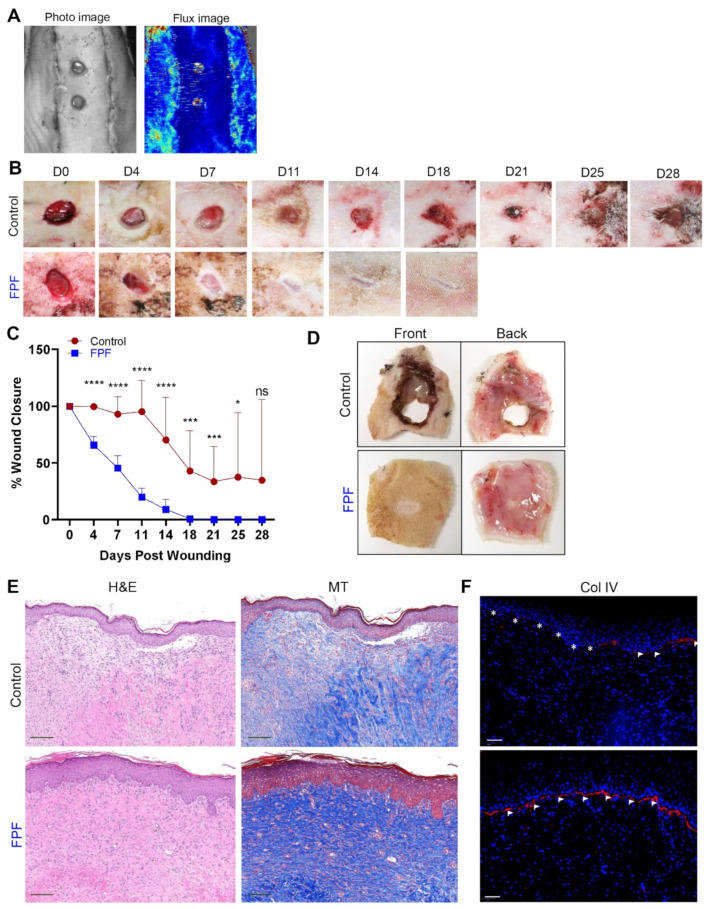
FPF accelerates wound closure in a rat ischemia wound model. (**A**) Representative photo image (left panel) and Doppler flux image (right panel), 10 min after completion of surgery, to confirm presence of ischemia in the bipedicle flap region. (**B**) Representative photographs of wounds taken over a 28-day period after wounding in control and FPF-treated groups. (**C**) Wound areas were measured and expressed as a percent of the wound area at day 0. Mean-SD for 5 rats per treatment group are shown for each time point on the graphs. *N* = 10, ns: not significant, * *p* < 0.05, *** *p* < 0.005, **** *p* < 0.001 FPF versus control. (**D**) Representative front and back photographs of necropsied tissue at day 28 after wounding for control and FPF-treated animals. (**E**) The structural integrity of healed tissue after wound closure in control and FPF-treated animals was assessed histologically by staining tissue sections with H&E (left panel) or MT (right panel). Scale bar = 5×, 192 μm (**F**) Basement membrane in the tissue samples collected after wound closure was visualized by collagen IV (Col IV) staining (red). Nuclei were counterstained with DAPI (blue). White asterisks highlight the absence of the basement membrane; white arrowheads highlight the basement membrane. Scale bar = 50 μm. *N* = 6/group.

Confirmation of tissue ischemia was achieved by use of a Doppler imager (Figure 4A). While the photo image (Figure 4A, left image) shows the creation of two wounds on the skin flap, the Doppler image (Figure 4A, right image) demonstrates a clear contrast in perfusion between the flap and the adjacent skin. The entire flap displayed a lack of blood supply to the flap from the incised edges, while the wounds were created in the ischemic section. Treatment with FPF resulted in total closure of ischemic wounds as early as 18 days after wounding, whereas control wounds remained open at 28 days after wounding (Figure 4B). Significant acceleration in the rate of ischemic wound healing was observed by the decrease in wound area of FPF-treated wounds as compared to the open control wounds (Figure 4C,D). Further, histological evaluation of FPF-treated wounds revealed proper granulation and re-epithelialization consistent with a normal rat dermis and epidermis in contrast to the control wounds that achieved closure (Figure 4E). The FPF-treated animals showed an appropriate level of tissue healing and the absence of epidermal hyperplasia and improper granulation tissue formation as were observed in the healed tissue sections of control animals (Figure 4E, left panel). Masson’s Trichrome staining further corroborated healthy deposition and maturation of collagen in FPF-treated wounds (Figure 4E, right panel). Furthermore, immunostaining for collagen IV, a component of the basal lamina, was continuous and well-formed in FPF-treated wounds but was tortuous and blunted in controls (Figure 4F). These results show that the factors present in FPF are conducive to pushing ischemic chronic wounds onto a path of regenerative tissue healing processes and achieve fitting wound closure. 

### 2.4. FPF Treatment Is Conducive for a Proangiogenic and an Anti-inflammatory Wound Microenvironment

**Figure 5:**
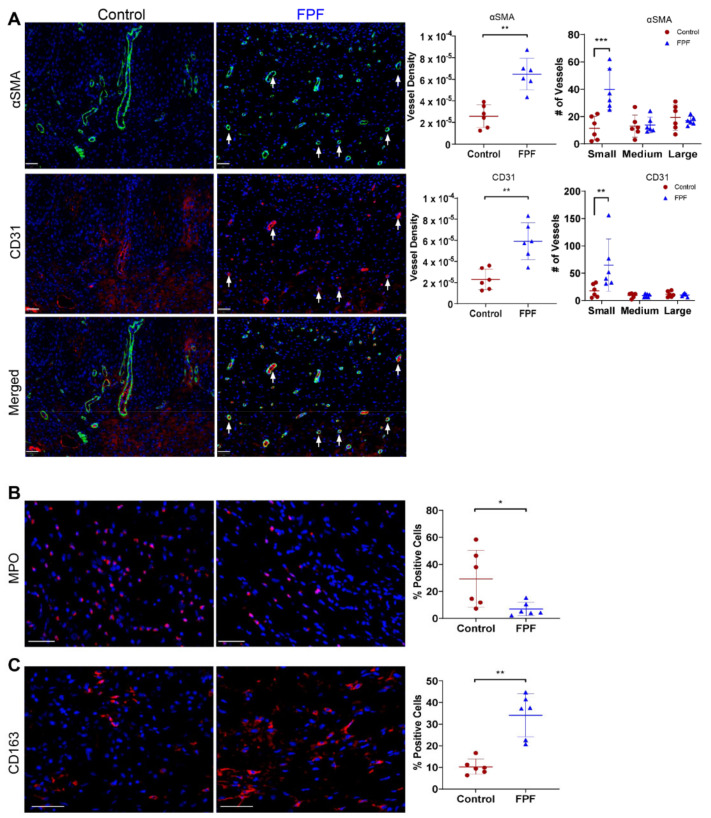
Angiogenic and anti-inflammatory response in FPF-treated wounds. (**A**) Neovascularization in control (left panel) and FPF treated (right panel) tissue samples collected after wound closure was determined by αSMA (top panel, stained green) and CD31 (middle panel, stained red). Merged images (bottom panel) show the colocalization of αSMA and CD31. Nuclei were counterstained with DAPI (blue). White arrows indicate αSMA- and CD31-positive blood vessels. Graphs represent the vessel density and size distribution of αSMA and CD31positive blood vessels. Scale bar = 50 μm. *N* = 6 for saline; *N* = 9 for FPF group. Inflammatory cell staining was determined by (**B**) MPO (red) staining for neutrophils. Scale bar = 50 μm and (**C**) CD163 (red) staining for M2 macrophages. Scale bar = 50 μm. Nuclei were counterstained with DAPI (blue). *N* = 6/group. * *p* < 0.05, ** *p* < 0.01, *** *p* < 0.005.

Ischemic wounds are primarily characterized by a reduction in neovascularization in the granulation tissue. To examine whether FPF supports tissue vascularization, we evaluated the healed tissue sections for blood vessel cell markers, namely smooth muscle actin and αSMA (Figure 5A, top panel), and an endothelial cell marker, CD31 (Figure 5A, middle panel). Both αSMA and CD31 were increased in FPF-treated wounds (Figure 5A, top and middle panel). The white arrows indicating αSMA- (Figure 5A) and CD31-positive (Figure 5B) blood vessels were representative of the formation of more blood vessels in FPF-treated wounds than in control wounds. CD31 stain colocalized in αSMA stained blood vessels (Figure 5A, bottom panel). Quantitative vessel density analysis for both αSMA and CD31 showed significant increases in the FPF-treated group as compared to controls (Figure 5A, vessel density). Furthermore, we evaluated the vessel results to visualize vessel size distribution by grouping vessels into small (<500 µm), medium (500–1000 µm), or large (>1000 µm) diameter. These data showed that FPF-treated wounds had a similar density of large or medium-sized blood vessels but a significantly greater density of small blood vessels compared to control wounds (Figure 5A, number of vessels). 

An ischemic microenvironment characterized by high levels of tissue hypoxia induces a proinflammatory phase. This results in exacerbated levels of inflammatory cells leading to impaired healing and in most cases chronic wounds. Tissue sections were stained for neutrophil marker myeloperoxidase (MPO) (Figure 5B) and M2 macrophage marker CD163 (Figure 5C). DAPI (blue staining) was used to visualize cell nuclei. We show that the FPF-treated animals present significantly lower levels of neutrophils as compared to the control healed wounds (Figure 5B). The influx of neutrophils in wound healing is followed by monocyte/macrophages. Although there was no significant difference between the levels of total macrophages (data not shown), we observed a significantly higher number of proresolving/anti-inflammatory M2 macrophages in the FPF-treated wounds (Figure 5C). Overall, our findings of the FPF-treated healed wounds suggest that accelerated and appropriate ischemic wound healing could be due to the observed changes in the wound microenvironment.

**Figure 6:**
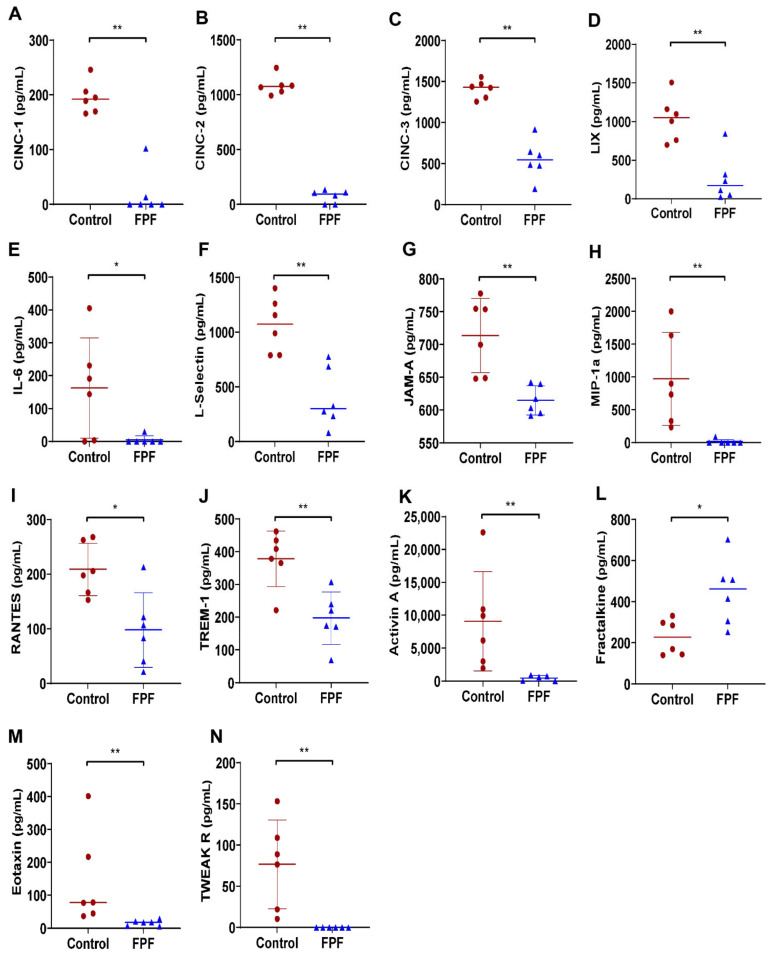
Anti-inflammatory effect of FPF on wound healing-associated cytokines. Levels of cytokines and chemokines in the wound tissue collected after wound closure for (**A**) CINC-1, (**B**) CINC-2, (**C**) CINC-3, (**D**) LIX, (**E**) IL-6, (**F**) L-selectin, (**G**) JAMA-A, (**H**) MIP-1α, (**I**) RANTES, (**J**) TREM-1, (**K**) activin A, (**L**) fractalkine, (**M**) eotaxin, and (N) TWEAK R. *N* = 6 rats/group. * *p* < 0.05, ** *p* < 0.01.

 Inflammatory cytokines/chemokines play a crucial role in the chemotaxis of proinflammatory cells. To elucidate the observed differences in inflammatory cells (Figure 6), we evaluated the cytokine/chemokine levels in the wound tissue by a multiplex signaling protein array (Figure 6). We show that FPF treatment significantly reduced the levels of important proinflammatory chemoattractants: cytokine-induced neutrophil chemoattractant-1 (CINC-1); cytokine-induced neutrophil chemoattractant-2 (CINC-2); cytokine-induced neutrophil chemoattractant-1 (CINC-3); lipopolysaccharide-inducible CXC chemokine (LIX); interleukin 6 (IL-6); L-selectin; junctional adhesion molecule A (JAM-A); macrophage inflammatory protein-α (MIP-1α); regulated upon activation, normal T cell expressed and presumably secreted (RANTES); triggering receptors expressed on myeloid cells-1 (TREM-1); activin A; eotaxin; tumor necrosis factor (TNF)-like weak inducer of apoptosis receptor (TWEAK R); and fractalkine (Figure 6A–N). Taken together, the observed consequences in FPF-treated wounds are suggestive of an anti-inflammatory granulation tissue microenvironment. 

### 2.5. FPF Induces Transcriptional Regulation Favorable for Ischemic Wound Healing

**Figure 7:**
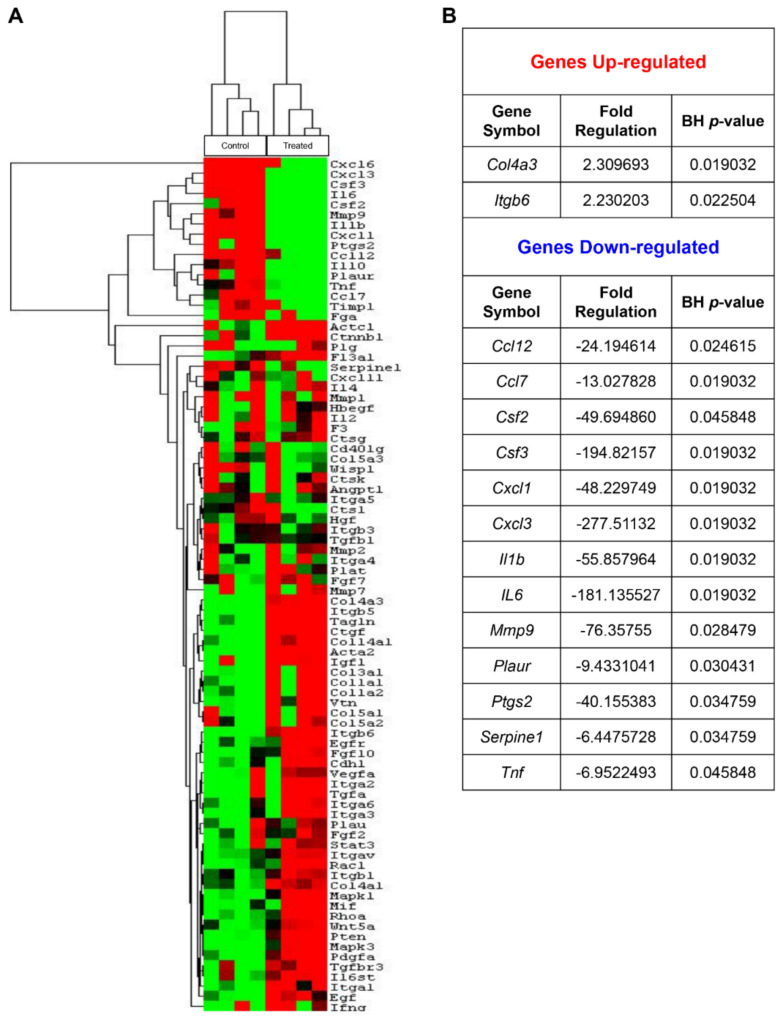
FPF treatment alters gene expression. Expression of 84 wound healing-associated genes in FPF-treated wounds was compared with that in control animals (**A**) Data are presented as a nonsupervised hierarchical clustering of genes with a heat map. (**B**) Individual rat wound healing genes with the most significant positive or negative change are shown for FPF-treated wounds vs. control wounds. *N* = 4/group. Benjamini-Hochberg adjusted *p*-value to increase stringency for RT^2^ PCR, where *p* < 0.05.

The complexity of nonhealing chronic wounds, largely in part due to the mixture of altered cytokine and growth factors in the wound bed, is orchestrated by misaligned transcription factors and dysregulated gene expression signatures. Gene array analysis of the collected healed tissue from both control and FPF-treated animals was performed to elucidate the wound healing gene signatures (Figure 7). The gene array included 84 rat wound healing-associated genes. The nonsupervised hierarchical clustering of genes with a heat map illustrates the fold change in genes expressed in control vs. FPF-treated wounds (Figure 7A). The criterion used to screen down- or upregulated genes was fold change of ≥2 and a Benjamini–Hochberg adjusted *p*-value ≤0.05 to increase strictness during panel profiling. Individual genes most significantly over- or under-expressed after FPF treatment are listed in the table (Figure 7B).

**Figure 8:**
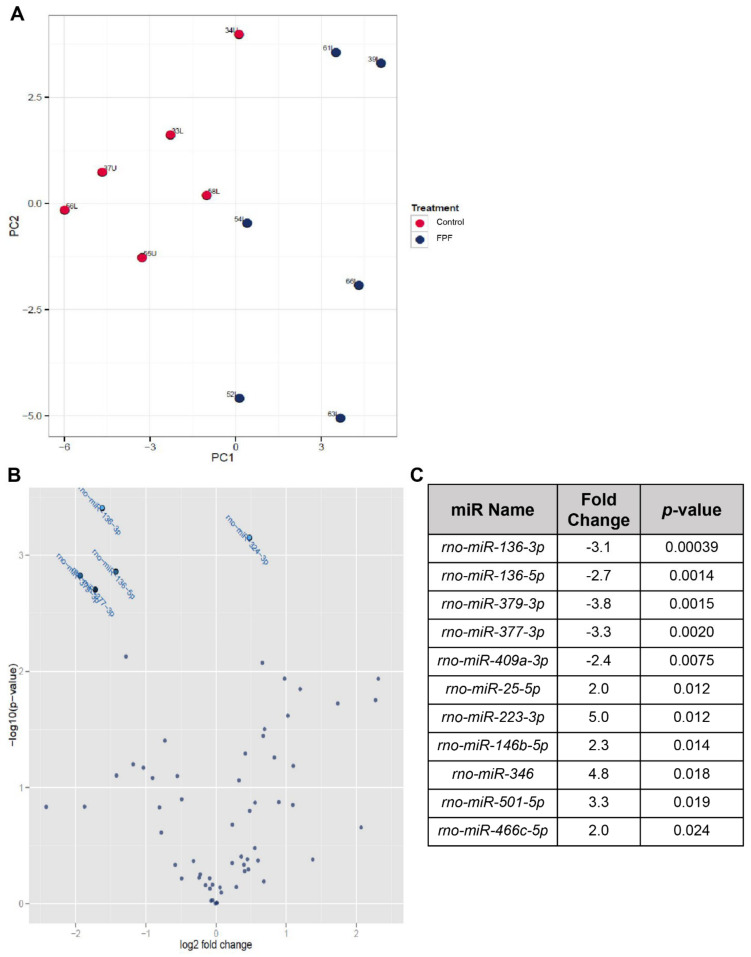
Transcriptional regulation is modulated by FPF treatment. MicroRNA profiling was performed on flash-frozen wound tissues using LNA-enhanced, SYBR Green based miRNA qPCR. (**A**) Principal component analysis (PCA) showing the distance and relatedness between samples with miRs having the largest coefficient of variation. (**B**) Volcano plot showing the relationship between p-values and fold change in normalized expression between control and FPF-treated groups. (**C**) Most differentially expressed known miRs with fold change (FC) between control and FPF-treated groups (*N* = 6/group).

Increasing evidence suggests that microRNA (miR) dysregulation leads to altered gene expression and outcomes in chronic wound pathogenesis and healing progression. To illuminate this possibility, we performed extremely specific and sensitive miRNA profiling using LNA-enhanced, SYBR Green based miRNA qPCR to identify and compare tissue-specific miR expression in FPF-treated and control ischemic wounds. A principal component analysis (PCA) was performed to identify two principal components (PC1 and PC2) (Figure 8A). The readily apparent independent clusters for control and FPF-treated groups were observed with the separation within the two groups (Figure 8A). Statistically significant differentially expressed miRs were identified from the constructed Volcano Plot (Figure 8B). The miRs with high statistical significance can be identified in two regions, the top of the plot and the left and right of the center of the plot. The criterion used to screen down- or upregulated miRs was fold change of ≥2 and a *p*-value ≤0.05 (Figure 8C). Based on these parameters, the most differentially downregulated known miRs are mir-136-3p, miR-136-5p, miR-379-3p, miR-377-3p, and miR-409a-3p. Upregulated differential expression was observed in miR-25-5p, miR-223-3p, miR-146b-5p, miR-346, miR-501-5p, and miR-466c-5p (Figure 8C). These differentially expressed miRs have been identified for their role in angiogenesis, regulation of inflammatory microenvironment, cell migration and apoptosis, reactive oxygen species generation, and restoring epithelial barrier function, all of which are altered in a chronic wound. Hence, the data suggest that FPF favorably modulates miRs that help in redirecting tissue healing conducive for appropriate restoration of the dermal tissue. 

## 3. Discussion

The field of regenerative medicine has been explored using various cells, cellular components, and biomaterials; however, translational progress and commercialization have been limited. The placental tissue as a biomaterial has been widely acknowledged to be a rich source of growth factors and cytokines, hyaluronic acid rich matrix, and endogenous viable cells, including MSCs [7,8]. Previous research has focused on the use of MSCs and methods to enhance the potential of these cells before use in stem cell-based therapies. To the best of our knowledge, there has been no work performed on preconditioning viable intact placental tissue as a whole with the embedded native cells and ECM and hence increasing the levels of endogenous growth factors present in the placental tissue [3]. This cocktail of factors holds tremendous tissue regenerative potential. Thus, the use of preconditioned placental tissue to generate placental tissue formulations (i.e., FPF) holds a wide assortment of therapeutic benefits. In this study, we first generated FPF using hypoxia primed placental tissue, followed by evaluating the beneficial characteristics of FPF in vitro, and then assessed FPF’s tissue regenerative potential in vivo in two separate rodent models of tissue injury. We show that FPF treatment increased tissue perfusion in a model of hindlimb ischemia, while controlling inflammation microenvironment in the surrounding muscle tissue. Additionally, in the bipedicle skin flap ischemic wound model, FPF-treated fast-healing wounds showed a reduction in inflammation, increase in angiogenesis, and overall improved tissue architecture. Furthermore, we observed gene and transcriptional changes in RT^2^ PCR and miRNA profiling qPCR analysis, respectively, in FPF-treated animals. 

A plethora of disease states and tissue injuries have inflammation- and ischemia-mediated pathophysiology [9,10]. Impaired healing in acute and chronic wounds exhibits high levels of IL-1 and TNF-α suggesting unresolved inflammation [11,12,13]. Nonhealing wounds, characterized by impaired cell proliferation and migration, are also marked by poor angiogenesis and thus display an ischemic microenvironment. Limb ischemia, caused by an obstruction in arterial blood flow to lower extremities and poor angiogenesis, is another condition that in severe cases progresses to chronic wounds. Due to the complexity of such diseases, therapeutic development using a multifactorial approach would be more effective. Our previous studies have indicated that the placental tissue (amnion, chorion, and umbilical cord), rich in cytokines, growth factors, and endogenous cells, provides both anti-inflammatory and angiogenic properties [14,15,16]. However, using hypoxia preconditioning, we show that endogenous properties of the placental membranes could be enhanced (Figure 1). Concentrations of both IL-1RA, a well-studied potent anti-inflammatory cytokine, and VEGF, a critical angiogenic growth factor, were increased when amniotic membranes and umbilical cord were primed under hypoxic conditions, suggesting an opportunity to further increase the reservoir of beneficial factors already contained in placental tissues [17,18]. During the priming process, the native viable placental cells, including MSCs, fibroblasts, and epithelial cells, are present in their natural microenvironment. Because the MSCs are not disrupted, their stemness and beneficial therapeutic properties could be preserved during the priming process. Furthermore, the ECM, while being a structural scaffold, is actively involved in providing signaling cues to cells and plays a dynamic role in cellular behavior to repair damaged tissue. Recent literature has shown the clinical use of placental tissue suspension in orthopedic indications [19]. However, the need for higher amounts of beneficial factors to meet the requirements to heal various disease indications might not be met by current combinatory placental formulations. An increase in endogenous growth factors, achievable by whole tissue priming, is needed to provide the concoction with heightened amounts of valuable factors. Thus, the primed viable placental membranes and umbilical cord, including their native ECM, viable native cells, growth factors, and other bioactive materials, as a whole, could be used in formulations that can be very effective in the various applications of regenerative medicine.

FPF is a rich microparticulate injectable cocktail of valuable cytokines, growth factors, and ECM matrix (Figure 2A) that are influential in alleviating chronic inflammatory and ischemic tissue environments. Since signaling molecules and growth factors are embedded in the placental ECM and cells within, the FPF configuration could result in a more sustained release of these factors. Studies have shown that the ECM is dynamically involved in host cellular interactions and provides signaling cues to repair the damaged tissue [20]. FPF was effective in both anti-inflammatory and angiogenic bioassays by inhibition of TNF-α and increasing the release of VEGF, respectively, suggesting that FPF could be functioning by modulating the inflammatory and angiogenic pathways. Using the in vivo limb ischemic model and an ischemic wound model, both with established increased inflammation and impaired vascularization, we showed that FPF was successful in tissue regeneration in both situations. This is suggestive that FPF functions via both the inflammatory and angiogenic pathways. Overall, these characteristics make FPF an attractive candidate for various therapeutic applications. 

Lower extremity ischemia due to decreased limb perfusion is the leading cause for amputations. The most prevalent cause of ischemia is progressive atherosclerotic stenosis of cardiac or limb arteries, which can be compounded by microvascular dysfunction in metabolic syndromes such as diabetes [21]. The increased generation of new vascular networks through the delivery of specific growth factors, MSCs, and umbilical cord-derived hydrogel are attractive strategies to restore perfusion to ischemic tissues and fill this unmet clinical need [22,23,24,25]. The observed significant increase in hindlimb perfusion upon FPF treatment provides evidence of its capability to reduce tissue ischemia (Figure 3B,C). Clinical research has also shown an increase in inflammatory biomarkers that predicts the presence of critical limb ischemia (CLI) [26]. Our data from the muscle tissue collected at 35 days postischemia indicated that treatment with FPF alleviated the inflammatory milieu that was otherwise observed in control hindlimbs (Figure 3D–G). The reduction in both human IL-8 homologs, CINC-1 and CINC-2, that induce the chemoattraction of neutrophils to an injury site suggests an anti-inflammatory role of FPF [27]. Similarly, levels of neutrophil and macrophage chemoattractant IL-1, also clinically observed in CLI patients, were significantly reduced in FPF-treated hindlimb ischemia animals [28]. The increase in tissue inflammation leads to an increase in RAGE, a proinflammatory pattern recognition receptor related to many inflammatory diseases. Further, RAGE binds to advanced glycation end products (AGEs) and promotes vascular inflammation in the blood vessels [29]. A significant decrease in RAGE observed in FPF-treated animals was further corroborated by the significant increases in anti-inflammatory, proangiogenic, and ischemia protective factors (Figure 3H–K). IL-22, increased upon FPF treatment, is reported to increase perfusion recovery and angiogenesis in ischemic conditions [30]. Furthermore, the occurrence of severe ischemia results in extensive damage and alterations to skeletal muscle [31]. IL-7, galectin-3, and erythropoietin, increased in FPF-treated animals, are all known to play a role in the regulation of muscle cell development and muscle tissue recovery after ischemic and traumatic tissue injury [32,33,34,35]. 

Since the principal observations from the hindlimb ischemic model suggested angiogenic properties of FPF, we next investigated FPF’s regenerative properties in a chronic ischemic rodent wound model (Figure 4). While localized tissue ischemia has been characterized as one of the major reasons for chronic wound development, incessant inflammatory microenvironments are classical biomarkers for chronic ischemic wounds [36,37]. By adopting an established and proven full-thickness bipedicle skin flap approach we were able to establish tissue ischemia, assessed by Doppler imaging, and created two full-thickness ischemic excisional wounds [36]. In order to aggravate impairment in ischemic wound healing, we increased the wound size to 8 mm. However, FPF treatment significantly improved both the rate and quality of wound closure (Figure 4B–F). Histologically, the presence of basket-weave-like structures indicated proper collagen deposition and maturity of the granulation tissue. Additionally, the presence of a well-established basement membrane demonstrated tight dermal–epidermal interactions in FPF-treated animals. Relatively higher expression of the Col4a3 gene, responsible for complex protein networks for basement membrane, further supported the occurrence of basement membrane in FPF-treated animals [38]. Our in vitro bioassay observation of FPF-induced VEGF production was supported in vivo by increased neovascularization, specifically small and newly formed vessels, suggested by dual αSMA and CD31 markers [39,40]. 

Similarly, our in vitro anti-inflammatory bioassay data was corroborated by in vivo improvement in inflammatory milieu observed in FPF-treated ischemic wounds (Figure 6 and Figure 7). The incessant and elevated presence in proinflammatory cytokines CINC-1, CINC-2, CINC-3, LIX, and IL-6, being crucial neutrophil traffickers, cause exacerbated levels of neutrophil incidence, resulting in further tissue damage [27,41,42,43]. Unlike FPF-treated wounds, control animals exhibited a significant increase in neutrophil chemoattractants that correlated with the upsurge in neutrophils to the wound bed (Figure 6B). Downregulation of genes, including Ccl12, Cxcl1, Cxcl3, IL-1β, IL-6, Ptgs2, and TNF, that contribute to inflammatory responses were observed in FPF-treated animals [44,45,46,47]. Furthermore, adhesion molecules L-selectin and JAM-A that are critical in neutrophil migration and activation were reduced upon FPF treatment. Both L-selectin and JAM-A heavily influence neutrophil extravasation and infiltration into the ischemic tissue environment [48,49,50]. Levels of eotaxin, which induces recruitment of not only eosinophils, but also basophils, neutrophils, and macrophages, were significantly reduced in FPF-treated animals [51]. We also saw significantly lower levels of MIP-1α, RANTES, TREM-1, and activin A, which are critical macrophage chemoattractants in wound repair [52,53,54,55]. TWEAK signaling further modulates inflammatory responses and enhances the production of proinflammatory cytokines, including RANTES [56]. While the expression of RANTES and its functional response to MIP-1α are enhanced during differentiation of monocytes to macrophages, activin A alters macrophage polarization by promoting a proinflammatory M1 phenotype and inhibiting the acquisition of anti-inflammatory M2 macrophage markers [55,57]. Although we did not find significant differences in the total number of macrophages, we detected a substantially higher number of M2 macrophages in FPF-treated animals (Figure 6C). Concurrently, we also found that FPF treatment led to an increase in fractalkine/CX3CL1, which induces VEGF-mediated angiogenesis potential in CX3CR1-expressing M2 macrophages [58]. 

Recent advances and understanding of miRs have proven their role in the regulation of gene expression. We thus analyzed the differences at the miR level using next-generation sequencing to evaluate the effect of FPF treatment on ischemic wounds. miRs are short conserved noncoding RNA molecules that regulate the translation of target mRNA and have been deemed crucial in various pathophysiological conditions. Dysregulation in different miRs has been acknowledged to cause deviations in crucial functions such as inflammation, angiogenesis, and cell migration and proliferation, leading to improper wound healing and chronicity [59]. As new targets for expression of various miRs have become validated, recent studies have reported a role for cells derived from the placental membrane and umbilical cord in positively affecting wound healing via miR modulation [60,61]. The inflammatory ischemic wound microenvironment combines increases in reactive oxygen species (ROS), cell death, and reduction in nitric oxide (NO) that regulates angiogenesis [62,63]. In our study, we observed that anti-inflammatory miR-346 responsible for inhibiting TNF-α release; miR-146-5p that reduces mRNA expression of IL-6 and IL-8; and miR-25-5p that suppresses cell apoptosis, generation of reactive oxygen species, and NO reduction were all upregulated in wounds treated with FPF [64,65,66]. Overexpression of miR-146b-5p has also been shown to protect during myocardial ischemia-reperfusion injury and to improve epithelial barrier function in intestinal injury [67,68]. On the contrary, downregulation of miR-136-5p, responsible for promoting the generation of inflammatory of IL-1β, IL-6, IFN-α, and TNF-α, was noted [69]. Moreover, miR-379 expression, associated with the epidermolysis bullosa severity (a severe skin disorder with extreme skin inflammation, epithelial fragility, and compromised barrier function), was reduced upon FPF treatment [70]. Likewise, miR-409a-3p, which has been reported to be expressed during the first week after spinal injury, was significantly reduced in FPF-treated animals [69]. Upregulation in miR-409a-3p also induces caspase 3 expression, causing fibroblast apoptosis and dermal scarring [71]. The expression of miR-136-3p, a distinguished clinical and in vivo marker in traumatic brain injury, was downregulated in our study [72]. The change in course from an inflammatory to an anti-inflammatory wound microenvironment is conducive to proper cell proliferation and migration, which could be supported by the observed increase in miR-501-5p [73]. Furthermore, miR-223-3p, the overexpression of which was shown to induce anti-inflammatory M2 macrophages in an LPS-induced sepsis model, was upregulated in FPF-treated animals and could likely contribute to our reported increase in M2 macrophages [74]. miR-223-3p also promotes angiogenesis of ischemic cardiac microvascular endothelial cells [75]. VEGF, being the principal angiogenic growth factor, is directly regulated by hypoxia-responsive miR-377-3p [76]. Knockdown of endogenous miR-377-3p not only promoted MSC-induced angiogenesis in ischemic hearts but also increased the vessel density significantly [76]. Consistent with these data, we report a significant decrease in miR-377-3p. 

In summary, for the first time, we report that the beneficial and endogenous growth factors of the placental membrane could be enhanced by hypoxia preconditioning/priming. In vitro results demonstrated FPF’s ability to modulate cell proliferation and migration and displayed both angiogenic and anti-inflammatory properties. Furthermore, the regenerative properties of FPF were supported by the data presented using two different in vivo models of ischemia tissue injury. While AM, CM, and UT have been clinically shown to support wound closure and improved re-epithelialization, our previous reports elucidate the underlying mechanisms for each individual tissue [14,15,16,77,78]. Thus, the observed benefits in both the hindlimb and ischemic wound model cannot be attributed to just one growth factor or single tissue; however, the combined effect of AM, CM, and UT in FPF possibly provides heightened tissue regenerative properties for therapeutic use. FPF is unique, as it consists of the amnion and chorion membranes and the umbilical cord. The collective therapeutic effects of FPF are led by multiple pathways, and hence they could potentially be expanded to various injury and disease indications.

## 4. Materials and Methods

### 4.1. Tissue Procurement, Processing, and Ethics Statement

Commercially available human full-term placentas were obtained from Cord Blood America, Inc. (CBA, Las Vegas, NV, USA) and the National Disease Research Interchange (NDRI, Philadelphia, PA, USA). Tissue procurement and ethics statement were provided by CBA and NDRI. Placentas were procured after obtaining written, informed consent from eligible donors. Placentas for this study were received and treated within 2 days. Human normal full-term placentas were processed as previously described [79]. Briefly, the viable amnion membrane tissue (AM) was separated from the viable chorion membrane tissue (CM) and umbilical cord (UC), cleaned, and treated with an antibiotic cocktail. The AM, CM, and UC were removed from the decidua and cleaned of all maternal tissue including blood and trophoblast. The UC was cleaned and large blood vessels were removed using blades and forceps [16]. Any residual blood from both UC and CM was removed by mechanically washing and cleaning with saline.

### 4.2. Preparation of Flowable Placental Formulation (FPF)

The cleaned AM, CM, and UC were stored in 250 mL of DMEM low glucose medium plus 0.5% HSA and primed at 37 °C at 2% O_2_ (hypoxia) plus 5% CO_2_ and 95% RH. After 48 h, the dishes were removed from the incubator and flowable placental formulation (FPF) was prepared using all AM, CM, and UC through a proprietary process, followed by lyophilization using Lyostar 2 lyophilization machine (SP Scientific, Lyostar 2, Gardiner, NY, USA). The lyophilized vials were irradiated and stored at room temperature prior to use. The lyophilized vials were resuspended in 1 mL saline solution immediately before use in in vitro and in vivo studies.

### 4.3. In Vitro Testing of Placental Membranes and FPF

Placental membrane (AM and CM) and UC priming, and growth factor analysis were completed as follows: AM, CM, and UC from three donors were cut into 3 × 4 cm dimension pieces using a scalpel and template. One 3 × 4 cm piece of each tissue was placed in a 10 cm cell culture dish and resuspended in 12 mL of DMEM + 2.5% FBS. Two sets were prepared, where one dish from each representative tissue was placed in a 37 °C incubator with 20% O_2_ (normoxia) and another dish was placed in a 37 °C incubator with 2% O_2_ (hypoxia). After 48 h, the dishes were removed from the incubator, and the tissue pieces were collected. Each 3 × 4 cm tissue piece was placed into a 2 mL Eppendorf tube and chopped finely using fine scissors. To the tube, 1 mL of T-PER (Thermo Fisher, Waltham, MA, USA) + protease inhibitor (Roche, St. Louis, MO, USA) was added to aid in tissue and cell lysis. To the tube, one 5 mm steel bead (Qiagen, Germantown, MD, USA) was added, and then the tube was placed in a TissueLyser LT (Qiagen, Germantown, MD, USA) and disrupted at maximum speed for 2 min, in three total cycles. The tube was then centrifuged at 14,000 rpm, and the supernatant was collected into another tube and frozen at −80 °C until assayed. The collected placental membrane lysates and supernatants were assayed using Multiplex (R&D Systems, Minneapolis, MN, USA) assay kit containing VEGF-A and IL-1RA. The assay was processed as per standard kit protocol and read using Luminex Magpix multiplex reader (Luminex, Austin, TX, USA). 

#### 4.3.1. Growth Factor Analysis of FPF

FPF was rehydrated with 1 mL of T-PER (Thermo Fisher, Waltham, MA, USA) + protease inhibitor (Roche, St. Louis, MO, USA). The tubes containing one 5 mm steel bead (Qiagen) were lysed for 2 min using the TissueLyser LT (Qiagen, Germantown, MD, USA). The tubes were then centrifuged at 14,000 rpm, and the supernatants were collected into other tubes and frozen at −80 °C until assayed. The collected supernatants were assayed using Multiplex (R&D Systems, Minneapolis, MN, USA) assay kit containing angiogenin, BMP-7, FGF-23, PDGF-AA, TIMP-1, VEGF-C, BMP-4, FGFb, osteopontin, PDGF-BB, VEGF-A, INF-gamma, IL-2, IL-6, TNF-alpha, IL-1beta, IL-5, CCL2/MCP-1, and IL-1RA. The assay was processed as per standard kit protocol and read using Luminex Magpix multiplex reader (Luminex, Austin, TX, USA). 

#### 4.3.2. VEGF Reporter Bioassay

VEGF reporter bioassay (Promega, Madison, WI, USA) was performed as per manufacturer instructions and read using a BioTek Synergy HTX plate reader. Briefly, the standard and FPF supernatants, as prepared earlier, were added to their respective wells, and then assay cells were thawed and added to each well. After 6 h of incubation, the luciferase substrate reagent was added to quantify the functional VEGF present in the media based on the standard curve. 

#### 4.3.3. TNFα Inhibition Bioassay

Lyophilized FPF was reconstituted in DMEM + 10% FBS and cocultured with 1 × 10^6^ PBMCs (AccuCell PBMCs Lot #13-090; Precision Bioservices, Frederick, MD, USA) and 1 µg/mL of LPS (Cat. L228; Sigma, St. Louis, MO, USA). PBMCs were treated with either LPS only or both LPS and FPF. Untreated PBMCs with both LPS and FPF were used as control. After 40 h of incubation in a 37 °C, 5% CO_2_ humidified incubator, cell suspensions were collected and centrifuged, and the TNF-α in the supernatant was quantified by ELISA (Cat. STA00D; R&D Systems, Minneapolis, MN, USA) according to manufacturer’s instructions.

#### 4.3.4. Preparation of FPF Conditioned Medium for the Scratch Wound Assay and MTT Assay

To perform a scratch wound assay and an MTT assay, 1 vial of FPF was rehydrated with 1 mL of Fibroblast Basal Medium (FBM) (Lonza, Walkersville, MD, USA) for 24 h in a 37 °C, 5% CO_2_ humidified incubator and then centrifuged at 3000 rpm for 20 min at room temperature. FPF conditioned medium was collected and used for the treatment of cells as the FPF group. FBM incubated and centrifuged under the same conditions was used as the control group. 

#### 4.3.5. Effect of FPF on the Migration of Human Dermal Fibroblasts (HDF) in Scratch Wound Assay

HDFs were plated at 5000 cells per well in silicon culture inserts (Ibidi, Integrated Bio Diagnostics, Munich, Germany) attached to 24-well tissue culture plates in FBM supplemented with FGM-2 SingleQuots (Lonza, Walkersville, MD, USA). The cells were gently washed with HBSS then incubated in FBM containing half of FGM-2 SingleQuots mixed with either an FBM (control group) or an FPF supernatant (FPF group) at the ratio of 1:1 (v/*v*) in a 37 °C, 5% CO_2_ humidified incubator. HDFs that migrated to the wounded area (initial diameter = 4 mm) the wound was imaged at days 0, 1, and 2 from the FPF supernatant treatment. Percent wound area was measured using ImageJ (NIH, Bethesda, MD, USA) in triplicate by three independent observers. 

#### 4.3.6. MTT Assay

Two thousand five hundred HDFs were grown per well in FBM containing half of FGM-2 SingleQuots mixed with either an FBM (control group) or an FPF supernatant (FPF group) at 37 °C for up to 4 days. Twenty microliters of MTT solution (Promega, Madison, WI, USA) per 100 μL of medium was added into cell cultures and incubated for 2 h at 37 °C. The absorbance of each well was measured at 490 nm using a plate reader. Data were obtained from at least three different experiments in quintuplicate.

### 4.4. In Vivo Testing of FPF

Animals: 12–14-week-old 400–500 g rats (Jackson Laboratories, Bar Harbor, ME, USA) were used in this study and housed at the Noble Life Sciences vivarium (Noble Life Sciences, Sykesville, MD, USA). Experimental protocols were approved by the Noble’s Institutional Animal Care and Use Committee. All procedures were performed in accordance with the guidelines and regulations of The Association for Assessment and Accreditation of Laboratory Animal Care International. All rats received Harlan Teklad Rodent Diet (Envigo, Madison, WI, USA) ad libitum. 

Hindlimb ischemia model: For hindlimb ischemia, the animals were anesthetized and maintained under anesthesia using isoflurane inhalation. The depth of anesthesia was assessed by lack of withdrawal reflex with toe pinch. Buprenorphine (analgesic, 5 mg/kg) and Baytril (5 mg/kg, antibiotic) were subcutaneously administered before the start of the surgery. The surgical site on the hindlimb was shaved and depilated to remove hair. Animals were placed on a heating pad to avoid hypothermia. Ophthalmic ointment/gel in both eyes of the animal was applied to prevent corneal desiccation. Sterile gauzes soaked in 70% ethanol were used to disinfect and clean the shaved region. Subcutaneous injection of 1% lidocaine was done along the incision site. For hindlimb ischemia, after the skin incision, the entire femoral artery (superficial, deep, and common femoral vessel) and all its major branches were ligated and excised. The external iliac artery and all of the above arteries were ligated with 3-5-0 silk suture and cauterized. Finally, the femoral artery was excised from its proximal origin as a branch of the external iliac artery, to the point distally where it bifurcates into the saphenous and popliteal arteries. As a consequence, blood flow to the ischemic limb becomes completely dependent upon collateral vessels issuing from the internal iliac artery. The incision site and skin layer were closed by a continuous suture using Ethibond suture 5-0. Postoperative analgesic buprenorphine, 5 mg/kg, was administered 6 h after initial injection on the operative day and twice a day for 2 additional days. Baytril, 5 mg/kg, was administered once a day for 5 days to prevent any infection. Confirmation of the creation in the ischemic bipedicle flap region was done by visualizing blood flow in the bipedicle flap and ischemic wounds with a High Resolution Laser Doppler Imager (Moor Instruments, Wilmington, DE, USA) before and after the ischemia procedure. Post-surgery images were acquired 10 min after the procedure. Rats were injected 1 day post-surgery with vehicle control (saline) or with flowable placental formulation (FPF) via intramuscular injection using a 22G needle (BD PrecisionGlide, Franklin Lakes, NJ, USA) on the same hindlimb that underwent ischemic surgery. Blood flow in both the hindlimbs was measured using a High Resolution Laser Doppler Imager (Moor Instruments, Wilmington, DE, USA) before the ischemia procedure and then on day 1 post-surgery. Following Doppler images were acquired on days 3, 7, 14, 21, 28, and 35 post-treatment. During Doppler imaging, the rats were anesthetized and maintained under anesthesia using isoflurane. At 5 weeks postischemia, all rats were euthanized and the Gracilis muscle was harvested for further histopathological and molecular analysis.

Dermal ischemic wound model: The ischemic bipedicle flap procedure was performed as described before with modifications [36]. The hair of animals on the back was removed by first using clippers (from the base of the neck down approximately 11 cm) and then with Nair lotion one day before the surgery. A sterile surgical ruler was used to outline the flap with a surgical marking pen (stencil with permanent marker, the outline for the 3.0 cm × 10.5 cm flap). Markings were made in the center of the flap at 5.0 cm from the top (centered along the spinal column and placed between the base of the scapulae and the iliac crest) to aid in wound placement. The rat’s dorsum was cleaned with alcohol and sprayed liberally with topical Betadine, and the entire surgery was performed under aseptic technique. Two adjacent 8 mm full-thickness excisional wounds were created in the vertical midline of the flap using a sterile 8 mm disposable biopsy punch. The depth of excision was down to, but did not rupture, the anterior fascia of the panniculus carnosus. The full-thickness punch biopsy, including skin and panniculus carnosus muscle, was removed by dissecting with scissors between the panniculus carnosus and the fascia. The dorsal bipedicle skin flap was raised in the craniocaudal direction deep to the panniculus carnosus muscle. Precut and sterilized nonreinforced 0.01 in. thickness Sil-Tec medical-grade sheeting was placed underneath the flap. The skin flaps and silicone sheet were sutured to the adjacent skin edges with interrupted nonabsorbable sutures to prevent movement of the silicone sheet. Tegaderm was applied to cover the entirety of the wound and bipedicle skin flap. Animals were left on the heating pad to recover from anesthesia. Postoperative analgesic buprenorphine, 5 mg/kg, was administered 6 h after initial injection on the operative day and twice a day for 2 additional days. Baytril, 5 mg/kg, was administered once a day for 5 days to prevent any infection. Rats were subcutaneously injected with 100 µL of either vehicle control (saline) or FPF using a 22G needle (BD PrecisionGlide, Franklin Lakes, NJ, USA) around the periphery of the wound on the day of surgery. 

Confirmation of the creation in the ischemic bipedicle flap region was done by visualizing blood flow in the bipedicle flap and ischemic wounds with a High Resolution Laser Doppler Imager (Moor Instruments, Wilmington, DE, USA) before and after the ischemia procedure. Doppler post-surgery image was acquired 10 min post-procedure. Photographic wound images were captured on days 0, 4, 7, 11, 14, 18, 21, 25, and 28, and wound areas were traced using ImageJ (NIH, Bethesda, MD, USA). During photographic and Doppler imaging, the rats were anesthetized and maintained under anesthesia using isoflurane. All rats were euthanized after wound closure, and the healed wound regions were harvested for further histopathological and flash-frozen for RNA analysis.

### 4.5. Histological and Immunohistochemical Assessment

Collected wound tissue samples were fixed in 10% neutral buffered formalin. Tissue sectioning and staining were performed by Premier Laboratory (Boulder, CO, USA) using standard protocols for hematoxylin and eosin (H&E), Masson’s trichrome (MT), myeloperoxidase (MPO), CD163, CD68, and α-smooth muscle actin (αSMA). H&E and MT slides were imaged using Aperio ScanScope AT2 (Leica Biosystems, Buffalo Grove, IL, USA), and immunofluorescent (IF) slides were imaged using Vectra 3 Automated Quantitative Pathology Imaging System (Akoya Biosciences, Marlborough, MA, USA) and assessed by a blinded independent pathologist. 

Samples for immunohistochemistry (IHC) were sectioned at 5 µm and mounted onto charged slides. Slides were dried overnight, baked at 60 °C for 1 h, deparaffinized in xylene, rinsed in alcohol, rehydrated in water, and equilibrated in wash buffer (TRIS buffered saline with 0.05% Tween 20; #K8007, Dako, Santa Clara, CA, USA). Heat-induced epitope retrieval (HIER) was performed in a Dako PT Link using a TRIS/EDTA buffer (FLEX TRS High, pH 9; #K8004, Dako, Santa Clara, CA, USA). A 20 min 95 °C retrieval was utilized prior to αSMA, CD31, and MPO staining. Slides were then cooled and rinsed in wash buffer, and the remaining IHC steps were carried out at room temperature on an Autostainer PlusLink stainer (Dako, Santa Clara, CA, USA). Proteolytic induced epitope retrieval (PIER) using Proteinase K for 5 min (S3020, Dako, Santa Clara, CA, USA) was performed prior to CD68, CD163, and collagen IV staining. Within the autostainer, a serum-free protein block was applied for 5 min (#X0909, Dako, Santa Clara, CA, USA)). 

The following primary antibodies and concentrations were used: for α smooth muscle actin (αSMA), rabbit polyclonal anti-αSMA primary antibody for 45 min (0.33 µg/mL) (#ab5694, Abcam, Waltham, MA, USA); for CD31, mouse monoclonal (TLD-3A12) anti-CD31 primary antibody for 45 min (10 µg/mL) (#MA1-80069, Invitrogen, Waltham, MA, USA); for CD68, mouse monoclonal (ED-1) anti-CD68 primary antibody for 30 min (5 µg/mL) (#MCA341, Biorad, Hercules, CA, USA); for CD163, mouse monoclonal (ED-2) anti-CD163 primary antibody for 30 min (6.67 µg/mL) (#MCA342, Biorad, Hercules, CA, USA); for collagen IV, rabbit polyclonal anti-collagen IV primary antibody for 60 min (10 µg/mL) (Invitrogen #PA1-28534); for MPO, rabbit polyclonal anti-MPO primary antibody for 30 min (1.33 µg/mL) (#ab9535, Abcam, Waltham, MA, USA). The following secondary antibodies and concentrations were used: for αSMA, goat anti-rabbit IgG Alexa Fluor 488 for 30 min (5 µg/mL) (#A11008, Invitrogen, Waltham, MA, USA); for CD31, CD68, and CD163, goat anti-mouse IgG1 Alexa Fluor 568 for 30 min (5 µg/mL) (#A21124, Invitrogen, Waltham, MA, USA); for collagen IV and MPO, goat anti-rabbit IgG Alexa Fluor Plus 555 for 40 min (6.67 µg/mL) ( #A32732, Invitrogen, Waltham, MA, USA). Wash buffer rinses were carried out between appropriate reagents. All slides were then manually counterstained for 5 min with DAPI (#D1306, Invitrogen, Waltham, MA, USA). The slides were rinsed in deionized water and coverslipped with Prolong Diamond Antifade Mountant (#P36970, Invitrogen, Waltham, MA, USA). For both αSMA and CD31 vessel density, all measurements were made using annotation tools in Indica Labs’ HALO software (Indica Labs, Albuquerque, NM, USA). Each vessel was manually measured using the ruler annotation tool. The vessels were measured at their widest point to capture the maximum diameter for each vessel. The total tissue area was measured using the pen annotation tool. The outside of the tissue was manually traced, and the area within this annotation was reported by HALO in square microns. Vessel density was calculated by dividing the total number of vessels by the total tissue area. Vessel size distribution for αSMA and CD31 was evaluated by grouping vessels into small (<500 µm), medium (500–1000 µm), or large (>1000 µm) diameter.

### 4.6. Cytokine/Chemokine Multiplex Analysis of Necropsied Animal Tissue Samples

Collected frozen samples of hindlimb Gracilis muscle and wound tissues were sent to RayBiotech (Peachtree Corners, GA, USA) for cytokine/chemokine analysis. Tissues were lysed and extracts were prepared according to RayBiotech’s standard tissue homogenization procedures. Tissues were lysed in the presence of T-PER (Thermo Fisher Scientific, Waltham, MA, USA) supplemented with a protease inhibitor cocktail (Sigma-Aldrich, St. Louis, MO, USA). Clarified supernatants, obtained after centrifugation at 14,000 rpm for 5 min, were analyzed for the presence of cytokines and chemokines using the Quantibody Rat Cytokine Array (Cat # QAR-CAA-67, RayBiotech, Peachtree Corners, GA, USA). The concentration of each analyte was normalized to the total protein concentration of the sample.

### 4.7. MicroRNA Array and PCR Gene Expression Analysis

Flash-frozen skin tissues collected after wound closure were sent to Qiagen Inc. (Germantown, MD, USA) for quantitative RT-PCR analysis (RT^2^ PCR) and microRNA (miR) qPCR analysis. RNA was isolated from flash-frozen tissues using the miRNeasy mini kit (Qiagen, Germantown, MD, USA) according to the manufacturer’s standard procedure (Qiagen, MD). RT^2^ PCR rat wound healing array was performed by Qiagen according to their standard protocol for Cat# PARN-121Z. For miR analysis, Qiagen used the extremely specific and sensitive LNA technology and the miRCURY LNA miRNA PCR system to run panel I+II for 752 miRs (Cat# YAMR-312Y).

### 4.8. Statistical Analysis

GraphPad InStat Software (GraphPad, La Jolla, CA, USA) was used for statistical analysis and plotting graphs. Results are presented as mean ± SD. Mann–Whitney test was used to determine the significance of differences between groups, whereby *p* < 0.05 was considered significant for Figure 1, Figure 2, Figure 3, Figure 4, Figure 5 and Figure 6. For Figure 7, a cutoff of *p*-value < 0.05 to pass a Benjamini–Hochberg correction was performed to increase stringency. Student’s t-test was used for Figure 8 comparing the control group to the FPF-treatment group where *p*-value < 0.05 was considered significant.

## Data Availability

The data presented in this study are available in the article.
